# Etiology and short-term outcome of pediatric coma at a tertiary hospital in Douala, Cameroon

**DOI:** 10.1186/s12887-025-06466-y

**Published:** 2025-12-30

**Authors:** Dominique Enyama, Soureya Haman, Fidèle Emmanuel Ngantchet, Corine Hwoguia Kamdem, Palma Haoua Abouame, Diomède Noukeu Njinkui, Joël Aquilas Ngalandeu Kwemo, Patrick Chrysologue Ngou Mfopou, Danièle Christiane Kedy Koum, Yacouba Njankouo Mapoure

**Affiliations:** 1 Faculty of Medicine and Pharmaceutical Sciences, University of Dschang, Dschang, Cameroon; 2 Pediatric Department, Douala Gyneco-Obstetric and Pediatric Hospital, P. O. Box 7270, Douala, Cameroon; 3 Faculty of Medicine and the Biomedical Sciences, University of Garoua, Garoua, Cameroon; 4 Faculty of Medicine and Pharmaceutical Sciences, University of Douala, Douala, Cameroon; 5 School of Health and Medical Sciences, Catholic University of Cameroon, Bamenda, Cameroon

**Keywords:** Coma, Children, Etiology, Outcome, Cerebral malaria, Glasgow coma scale, Mortality, Sub-Saharan Africa

## Abstract

**Background:**

Pediatric coma is a critical emergency with high morbidity and mortality in sub-Saharan Africa, where limited data hinders effective management strategies. Understanding its epidemiology and prognostic factors is essential for improving outcomes.

**Methods:**

A cross-sectional study with retrospective (1st January 2017 to 30 November 2018) and prospective (1st December 2018 to 30th April 2019) phases was conducted at Gyneco-Obstetric and Pediatric Hospital of Douala, Cameroon. Children aged 1 month to 15 years with Glasgow Coma Scale (GCS) ≤ 14 were included. Data on demographics, clinical presentation, etiology, and outcomes were collected. Statistical analysis used SPSS version 20.0 and CSPro with Chi-square, Fisher’s exact tests, and multivariable logistic regression.

**Results:**

Among 864 hospitalized children, 109 presented with coma (prevalence 12.6%), comprising 88 retrospective and 21 prospective cases. The male-to-female ratio was 1.4:1, with mean age 48.8 ± 47.5 months; 64.2% were under 5 years. Infectious causes predominated (62.4%, *n* = 68), with cerebral malaria accounting for 42.2% (46/109) and septicemia 15.6% (17/109). Other etiologies included metabolic/toxic causes (16.5%, 18/109), post-epileptic coma/status epilepticus (14.7%, 16/109), and traumatic brain injury (4.6%, 5/109); 12.8% (14/109) remained undiagnosed. Clinical features included fever (73.4%, 80/109) and seizures at admission (68.8%, 75/109). Overall mortality was 26.6% (29/109), with 30.9% (25/81) of survivors experiencing neurological sequelae, predominantly motor deficits (14.8%, 12/81). In multivariable analysis, significant mortality predictors included age under 2 years (adjusted OR 4.55, 95% CI: 1.23–16.82), female sex (adjusted OR 2.89, 95% CI: 1.23–6.79), direct home admission (adjusted OR 2.76, 95% CI: 1.02–7.47), and deeper coma stages (Stage III-IV: adjusted OR 6.92, 95% CI: 2.54–18.86).

**Conclusion:**

Pediatric coma at this tertiary center in Douala predominantly affects young children and stems primarily from infectious etiologies, particularly cerebral malaria. The high mortality (26.6%) and substantial neurological morbidity among survivors underscore urgent needs for strengthened malaria prevention programs, improved community awareness, enhanced referral systems, and increased diagnostic and intensive care capabilities. Early recognition and prompt management of preventable causes could significantly reduce mortality and morbidity from pediatric coma in Central Africa.

**Supplementary Information:**

The online version contains supplementary material available at 10.1186/s12887-025-06466-y.

## Introduction

Coma is defined as a prolonged loss of consciousness resulting from dysfunction of the ascending reticular activating formation (ARAF), which is responsible for arousal and maintenance of wakefulness [1–4]. In pediatric practice, coma represents a critical emergency, accounting for 10–15% of all hospitalizations and associated with substantial morbidity and mortality [5–7].

The epidemiology of pediatric coma varies significantly across geographic regions. In England, the incidence of non-traumatic coma has been estimated at 30.8 per 100,000 children under 16 years annually, with etiology-specific mortality rates ranging from 3% to 84% [8]. In Saudi Arabia, Ali et al. reported an incidence of 4.8 per 100,000 children per year for both traumatic and non-traumatic coma, with a mortality rate of 47.2% [9]. African studies have shown variable prevalence rates: 5.4% in Lomé, Togo (mortality 27.6%) [3], and 14.4% in Cairo, Egypt (mortality 50%) [1].

In sub-Saharan Africa and Asia, the predominant etiologies of pediatric coma are infections of the central nervous system, including cerebral malaria, acute bacterial meningitis, and viral encephalitis. Understanding the etiology and predictive factors of coma outcomes is crucial for developing improved management strategies, particularly in resource-limited settings where diagnostic and supportive systems are rarely available [10].

The prognosis of coma depends on both etiology and the rapidity of appropriate management [11–13]. Outcomes range from complete recovery to persistent neurological sequelae or death.

In Douala, Cameroon’s largest city and economic capital, limited data exist on pediatric coma. This study was therefore undertaken to describe the etiologies and determine the short-term outcomes of childhood coma at the Douala Gyneco-Obstetric and Pediatric Hospital (DGOPH), a major tertiary referral center.

## Materials and methods

### Study design, area, and population

We conducted a cross-sectional study to determine the prevalence, etiology, and short-term outcomes of pediatric coma at the DGOPH in Cameroon. The study incorporated two complementary data collection approaches: (1) Retrospective component with systematic review of medical records for children presenting with coma between January 2017 and November 2018 (88 cases identified) and (2) Prospective component with a real-time enrollment and standardized data collection for children presenting with coma between December 2018 and April 2019 (21 cases enrolled).

This cross-sectional study spanned 28 months (January 2017–April 2019) with active data collection and analysis from December 2018 to June 2019. To ensure an adequate sample size for the relatively rare condition of pediatric coma, a dual-phase approach was used: a retrospective review of 88 medical records (January 2017–November 2018) and prospective enrollment of 21 cases (December 2018–April 2019), totaling 109 patients for analysis.

The study was conducted at DGOPH, a major tertiary referral hospital in Douala, Cameroon, serving a population of approximately 3 million. Its pediatric service included general pediatrics, neonatology, and an outpatient unit, staffed by pediatricians, general practitioners, and paramedical personnel. Available resources included a clinical laboratory, video-EEG, and CT imaging, though intensive care capabilities were limited to a high-dependency area within the general ward, two pediatric ventilators, and basic monitoring and respiratory support.

The study population consisted of children aged 1 month to 15 years admitted with coma, defined as a Glasgow Coma Scale (GCS) score ≤ 14. Inclusion required a GCS ≤ 14, adequate records (retrospective), or informed consent (prospective). Neonates under one month and cases with incomplete records or lacking consent were excluded. A sample size calculation, based on a 5.4% estimated prevalence, determined a minimum of 79 patients; the final sample of 109 provided sufficient statistical power.

### Data collection procedures

Following ethical approval from institutional committees, data collection was conducted in two phases. The retrospective phase entailed a systematic review of hospital admission registers and medical records to identify eligible pediatric coma cases (GCS ≤ 14) from January 2017 to November 2018. For the prospective phase from December 2018 to April 2019, consecutive eligible patients whose parents provided consent were enrolled. In both phases, data were captured using standardized case report forms, and each prospectively enrolled child received a systematic clinical examination, including a detailed neurological assessment and GCS scoring by trained physicians.

### Variables and definitions

#### Sociodemographic and clinical variables

Sociodemographic data collected included age, sex, city of residence, and referral source (directly from home vs. referred from another healthcare facility). Medical history captured psychomotor development status, previous episodes of coma, seizure history, known epilepsy diagnosis, and ongoing antiepileptic treatment.

The collected data encompassed detailed clinical, laboratory, and imaging variables. Clinical characteristics recorded at admission included vital signs, neurological status using the Glasgow Coma Scale (GCS), coma stage, and a systematic assessment for associated symptoms and signs. Laboratory investigations targeted infectious, metabolic, and hematologic parameters. Neuroimaging and electrophysiological studies were utilized to evaluate structural and functional brain abnormalities.

#### Operational definitions

- *Coma *was defined as a Glasgow Coma Scale score ≤ 14 out of 15, using either the standard Glasgow Coma Scale for verbal children or the pediatric Glasgow Coma Scale for pre-verbal children. The rationale for the GCS ≤ 14 threshold was to capture all patients with clinically significant altered consciousness requiring emergency intervention and intensive monitoring. While GCS ≤ 8 represents severe coma in many studies, we included patients with GCS 9–14 because: (1) in resource-limited settings, even moderate consciousness impairment requires intensive observation due to limited monitoring capabilities; (2) these patients remain at risk for rapid deterioration; (3) previous studies from similar settings have used comparable thresholds; and (4) this approach allows better characterization of the full spectrum of consciousness disorders in our population.
- *Coma severity was staged based on GCS scores*: Stage I (Vigil Coma) for GCS 11–13, Stage II (Light Coma) for GCS 9–10, Stage III (Deep Coma) for GCS 6–8, and Stage IV (Beyond Coma/Carus) for GCS 3–5.
- *Temperature definitions were fever*: ≥38 °C (measured by axillary or rectal thermometry), normothermia: 36.5–37.9 °C, and hypothermia: <36.5 °C.
- *Glucose definitions based on capillary measurement were hypoglycemia*: ≤0.50 g/L (≤ 2.8 mmol/L), normoglycemia: 0.51–1.25 g/L (2.9–6.9 mmol/L), and hyperglycemia: ≥1.26 g/L (≥ 7.0 mmol/L).
- *Severe pallor* clinically assessed using palmar and conjunctival examination.
- *Respiratory distress,* defined as tachypnea, increased work of breathing, or oxygen desaturation < 90%.
- *Seizures*, defined as witnessed convulsive activity or documented seizures in medical records.

#### Etiological definitions

The following standardized criteria were used for etiological classification.

- *Cerebral malaria:* Positive *Plasmodium falciparum* parasitemia, GCS ≤ 14, exclusion of other encephalopathy causes, with supportive features like seizures, hypoglycemia, or severe anemia.
- *Septicemia/sepsis:* Systemic infection signs (fever/hypothermia, tachycardia, tachypnea), evidence of organ dysfunction, with or without positive blood culture, and no other primary infection site.
- *Bacterial meningitis:* CSF pleocytosis plus positive CSF Gram stain/culture or positive blood culture with compatible CSF findings, accompanied by clinical signs.
- *Herpes encephalitis*: Encephalitis symptoms, CSF lymphocytosis (if available), characteristic neuroimaging, and response to acyclovir.
- *Febrile gastroenteritis with dehydration*: Diarrhea/vomiting, clinical dehydration, fever, coma attributed to dehydration/electrolyte disturbance, with CNS infection excluded.
- *Metabolic causes*: Includes hyponatremia (< 130 mmol/L), hypoglycemia (≤ 0.50 g/L), or diabetic ketoacidosis (hyperglycemia > 11 mmol/L with ketones and acidosis).
- *Intoxication*: History of toxic exposure, compatible symptoms, exclusion of other causes, and response to supportive care/antidote.
- *Post-epileptic coma*: Witnessed seizures, GCS ≤ 14 persisting > 30 min post-ictal, known or first-time epilepsy, with acute CNS infection/structural lesion excluded.
- *Status epilepticus*: Continuous seizures ≥ 30 min or recurrent seizures without consciousness recovery for ≥ 30 min.
- *Traumatic brain injury*: Clear head trauma history, consistent neurological signs, and supportive CT findings when available.
- *Unknown etiology*: No identifiable cause after thorough evaluation, with negative tests for infection, metabolic abnormalities, trauma, and ingestion.

#### Outcome definitions

The primary short-term outcome was patient status at hospital discharge. Status categories were in-hospital mortality, survival without neurological sequelae, survival with neurological sequelae, discharge against medical advice (DAMA), transfer to another facility, or ICU admission. Neurological sequelae were defined as new deficits or functional deterioration present at discharge compared to pre-morbid baseline.

A comprehensive, multi-domain assessment for sequelae was conducted through structured clinical exams and parental interviews. This included evaluating motor function (graded by severity and type), visual and hearing acuity (via bedside and interview methods, as formal audiometry was unavailable), psychomotor development (using milestone checklists to identify regression), and new behavioral disorders. All assessments were performed and documented by pediatricians. The authors note the limitations of this clinical assessment for detecting subtle or delayed deficits, which would require longer follow-up. Hospital stay duration was also recorded in predefined categories.

### Data analysis

Data were entered using Census and Survey Processing System (CSPro) version 7.1 and analyzed using SPSS version 20.0 (IBM Corp., Armonk, NY, USA). Tables and figures were created using Microsoft Excel 2010.

#### Descriptive statistics

Categorical variables were summarized as frequencies and percentages with explicit denominators. Continuous variables were described using means with standard deviations (SD) for normally distributed data, or medians with ranges for skewed distributions. Normality was assessed using Shapiro-Wilk tests and visual inspection of histograms.

#### Handling of missing data

Missing data were handled using a complete-case analysis approach, without imputation, due to data being missing not at random as a result of resource limitations. For all analyses, the relevant denominator (either the total cohort or the subset tested) is explicitly stated. Patients with missing data for the variables in a given comparison were excluded from that specific analysis. Variables with more than 30% missingness were omitted from multivariable models. The potential impact of missing data patterns on key conclusions was evaluated through sensitivity analyses.

#### Comparative and multivariable analyses

Statistical analyses were conducted using univariate and multivariable methods. Univariate comparisons employed Chi-square, Fisher’s exact, t‑tests, or Mann‑Whitney U tests as appropriate. To identify independent predictors of mortality, a multivariable logistic regression model was constructed. Clinically relevant variables with a univariate p‑value < 0.20 and < 30% missing data were considered. The final model, built via forward stepwise selection, included age group, sex, referral source, and GCS stage. Model diagnostics confirmed adequate fit, absence of multicollinearity (VIF < 2.5), and good discrimination. Results are reported as odds ratios with 95% confidence intervals, with significance at *p* < 0.05. A sensitivity analysis examined outcomes using alternative GCS thresholds.

## Results

### Study population and patient flow

**Fig. 1:**
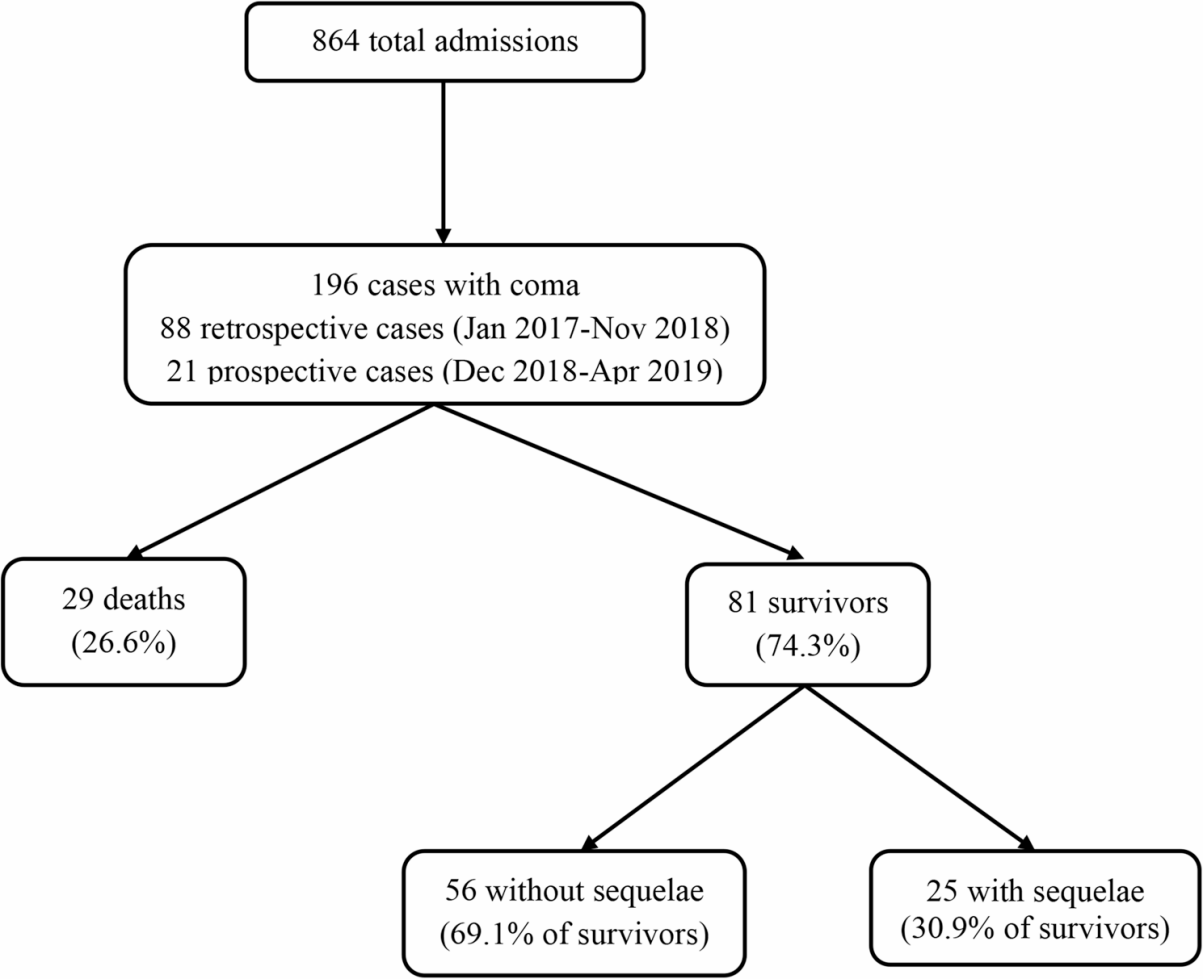
Patient flow diagram

During the 28-month patient inclusion period (January 2017 to April 2019), 864 children aged 1 month to 15 years were hospitalized at DGOPH. Of these, 109 (12.6%) presented with coma (GCS ≤ 14), comprising 88 cases (80.7%) identified through retrospective record review and 21 cases (19.3%) enrolled prospectively. (See Fig. 1). All 109 cases were included in the analysis.

Patient outcomes included 29 deaths (26.6%) and 81 survivors (74.3%). Among survivors, 56 (69.1% of survivors; 51.4% of total) were discharged without sequelae and 25 (30.9% of survivors; 22.9% of total) with sequelae. Other outcomes were discharge against medical advice (6 patients, 5.5%), transfer to other facilities (3 patients, 2.8%), and requirement for mechanical ventilation/ICU admission (2 patients, 1.8%).

### Sociodemographic characteristics

Males constituted 57.8% (63/109) of the cohort, giving a male-to-female ratio of 1.4:1. The mean age was 48.8 ± 47.5 months (median: 36 months; range: 1-180 months). Children under 5 years represented nearly two-thirds (64.2%, 70/109) of all cases. Male predominance was most pronounced in adolescents (85.7%) compared to infants under 3 months (25.0%). (See Table 1)

**Table 1:** Age and sex distribution of children with coma (*N* = 109)

Age Group	Male (%)		Female (%)		Total (%)	
1–3 months	1 (25.0)	3 (75.0)		4 (3.7)		
3–24 months	24 (51.1)	23 (48.9)		47 (43.1)		
25–59 months	10 (55.6)	8 (44.4)		18 (16.5)		
5–10 years	16 (64.0)	9 (36.0)		25 (22.9)		
10–15 years	12 (85.7)	2 (14.3)		14 (12.8)		
Total	63 (57.8)	46 (42.2)		109 (100)		

The geographic distribution of patients showed that the majority, 100 (91.7%), resided in Douala city, while 5 (4.6%) came from Buea and 4 (3.7%) from other cities such as Kribi and Edéa. Regarding referral source, most patients, 81 (74.3%), were referred from other healthcare facilities, with the remaining 28 (25.7%) brought directly from home.

### Medical history

Regarding developmental status, 100 patients (91.7%) had normal psychomotor development while 9 (8.3%) had abnormal development. A history of previous seizures was present in 19 patients (17.4%), previous coma in 2 (1.8%), known epilepsy in 6 (5.5%), and 6 (5.5%) were on antiepileptic medication at admission.

### Clinical presentation

Coma severity at admission (see Table 2) was as follows: Stage I (Vigil Coma, GCS 11–13) in 26 patients (23.9%), Stage II (Light Coma, GCS 9–10) in 49 (45.0%), Stage III (Deep Coma, GCS 6–8) in 22 (20.2%), Stage IV (Beyond Coma, GCS 3–5) in 11 (10.1%), and stage not documented in 1 (0.9%). The majority of patients presented with Stage II coma (45.0%, 49/109). Temperature at admission showed fever (≥ 38 °C) in 80 patients (73.4%), normothermia (36.5–37.9 °C) in 20 (18.3%), and hypothermia (< 36.5 °C) in 9 (8.3%). Associated clinical findings were seizures at admission in 75 patients (68.8%), of which 64 (85.3%) were generalized and 11 (14.7%) focal; respiratory distress in 50 (45.9%); digestive disorders in 42 (38.5%); severe pallor in 25 (22.9%); skin rash in 12 (11.0%); ENT infections in 7 (6.4%); and clinical malnutrition in 6 (5.5%).

**Table 2:** Clinical characteristics at admission (*N* = 109)

Clinical Feature	Number, *n* (%)
Coma Severity (Glasgow Coma Scale)	
Stage I (GCS 11–13)	26 (23.9)
Stage II (GCS 9–10)	49 (45.0)
Stage III (GCS 6–8)	22 (20.2)
Stage IV (GCS 3–5)	11 (10.1)
Temperature	
Fever (≥ 38 °C)	80 (73.4)
Normothermia (36.5–37.5 °C)	20 (18.3)
Hypothermia (< 36.5 °C)	9 (8.3)
Associated Features	
Seizures at admission	75 (68.8)
Respiratory distress	50 (45.9)
Digestive disorders	42 (38.5)
Severe pallor	25 (22.9)
Skin rash	12 (11.0)
ENT infection	7 (6.4)
Malnutrition	6 (5.5)

### Laboratory and imaging investigations

Diagnostic investigation completion rates and reasons for missing data (*N* = 109) were as follows: Capillary glucose was performed in 107 patients (98.2%), with the main reason for non-completion being rapid death before measurement. Malaria testing was completed for 97 (89.0%), with non-completion due to clinical diagnosis being obvious or rapid death. Hemoglobin was measured in 105 (96.3%), with non-completion due to equipment unavailability or rapid death. Serum sodium was tested in 49 (45.0%), with cost limitations and clinical judgment as main reasons for the 60 tests not performed. C-reactive protein was tested in 75 (68.8%), with cost and availability as reasons for the 34 tests not done. Lumbar puncture was performed in 34 (31.2%), with contraindications, refusal, or rapid improvement preventing it in 75 cases. Brain CT scan was done for 24 (22.0%), with cost (not insurance-covered) and limited availability as reasons for the 85 not performed. Brain MRI was performed for 5 (4.6%), with high cost and very limited availability preventing it in 104 cases. EEG was done for 9 (8.3%), with limited availability and equipment issues preventing it in 100 cases. Blood culture was performed for 18 (16.5%), with cost and limited laboratory capacity as reasons for the 91 not done.

Laboratory findings were as follows: For glucose homeostasis (*n* = 107 tested), hypoglycemia (≤ 0.50 g/L) was found in 13/107 (12.1%), normoglycemia in 84/107 (78.5%), and hyperglycemia (≥ 1.26 g/L) in 10/107 (9.3%). For malaria testing (*n* = 97 tested), 46/97 (47.4%) were positive (thick smear or RDT) and 51/97 (52.6%) were negative. For serum sodium (*n* = 49 tested), hyponatremia (< 130 mmol/L) was found in 13/49 (26.5%) and normal sodium in 36/49 (73.5%); the overall prevalence of documented hyponatremia was 13/109 (11.9%). For hemoglobin (*n* = 105 tested), the mean was 9.8 ± 3.2 g/dL, with severe anemia (Hb < 5 g/dL) in 8/105 (7.6%), moderate anemia (Hb 5–9.9 g/dL) in 51/105 (48.6%), and mild anemia or normal (Hb ≥ 10 g/dL) in 46/105 (43.8%). C-reactive protein (*n* = 75 tested) was elevated (> 10 mg/L) in 68/75 (90.7%). For cerebrospinal fluid analysis (*n* = 34 performed), 30/34 (88.2%) were normal, bacterial meningitis was confirmed in 1/34 (2.9% of those tested; 0.9% of total cohort), CSF culture was positive in 1/34 (2.9%), and viral/aseptic meningitis was suspected in 3/34 (8.8%). Neuroimaging results showed brain CT was performed in 24/109 (22.0%) patients, with abnormal findings in 12/24 (50.0%) and normal results in 12/24 (50.0%). Brain MRI was performed in 5/109 (4.6%) patients, and all showed abnormalities (temporal lobe changes in herpes encephalitis cases). For electroencephalography, EEG was performed in 9/109 (8.3%) patients, with abnormal results in 7/9 (77.8%) and normal results in 2/9 (22.2%).

### Etiological distribution

**Fig. 2:**
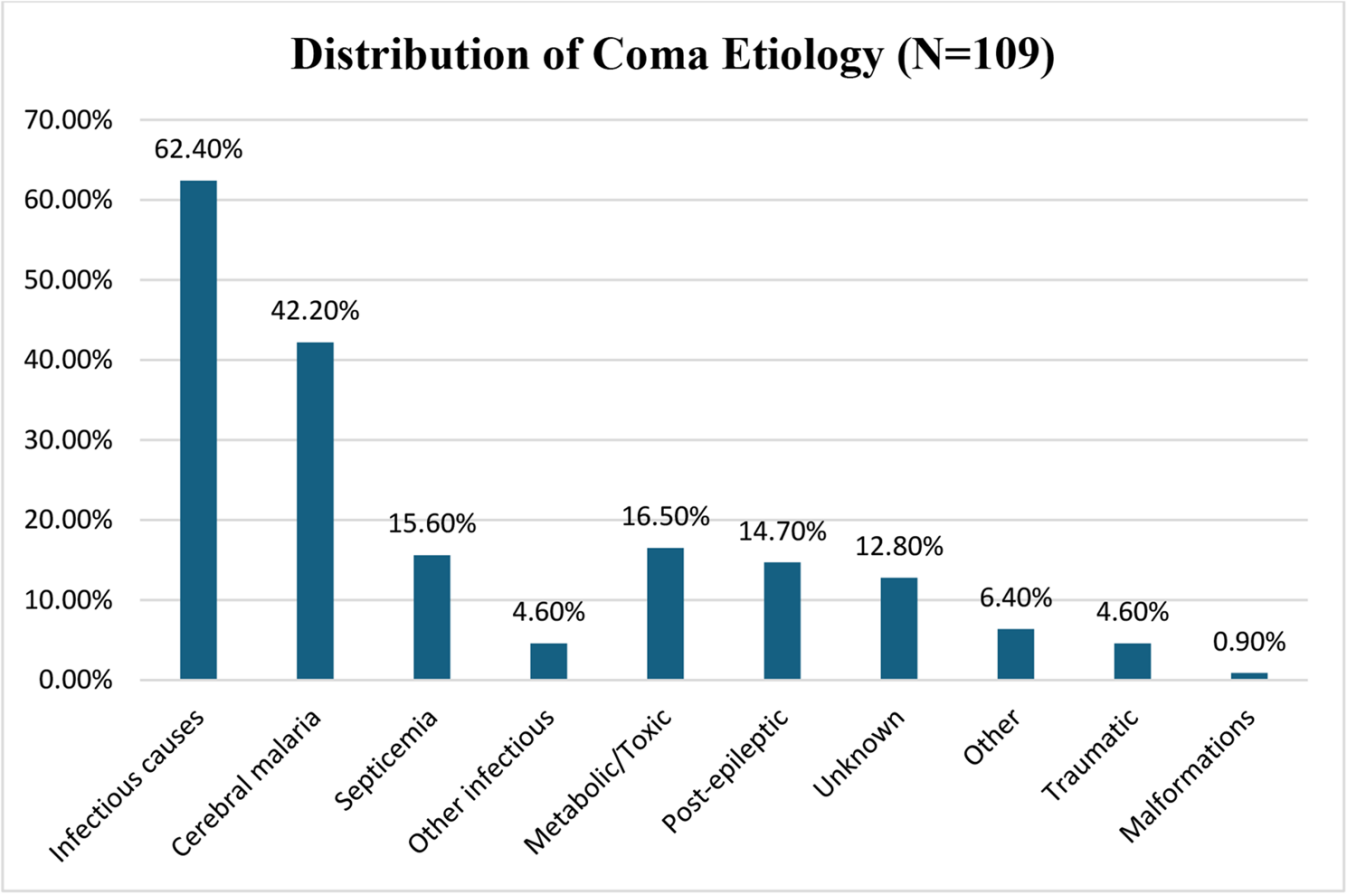
Distribution of coma etiology (N=109)

The etiological distribution of pediatric coma (*N* = 109) was as follows. Infectious causes predominated with 68 cases (100% of the category; 62.4% of total). This category included cerebral malaria (46 cases, 67.6% of category, 42.2% of total), septicemia (17 cases, 25.0% of category, 15.6% of total), febrile gastroenteritis with dehydration (7 cases, 10.3% of category, 6.4% of total), herpes encephalitis (3 cases, 4.4% of category, 2.8% of total), and bacterial meningitis (1 case, 1.5% of category, 0.9% of total). Figure 2 illustrates the relative proportions of different etiological categories.

Metabolic/Toxic causes accounted for 18 cases (100% of category; 16.5% of total), including hyponatremia (13 cases, 72.2% of category, 11.9% of total), hypoglycemia (3 cases, 16.7% of category, 2.8% of total), intoxication (petroleum, medications) (3 cases, 16.7% of category, 2.8% of total), and diabetic ketoacidosis (1 case, 5.6% of category, 0.9% of total). Post-epileptic/Status epilepticus accounted for 16 cases (100% of category; 14.7% of total), including status epilepticus (9 cases, 56.3% of category, 8.3% of total) and post-epileptic coma (7 cases, 43.8% of category, 6.4% of total). Traumatic brain injury accounted for 5 cases (100% of category; 4.6% of total). Other causes accounted for 7 cases (100% of category; 6.4% of total), including congenital malformations (1 case, 14.3% of category, 0.9% of total) and others/mixed (6 cases, 85.7% of category, 5.5% of total). Unknown etiology accounted for 14 cases (12.8% of total) See Table 3.

**Table 3:** Etiological distribution of pediatric coma (*N* = 109)

Etiology	Number (%)	Deaths (%)
Infectious causes	68 (62.4)	19 (27.9)
Cerebral malaria	46 (42.2)	9 (19.6)
Septicemia	17 (15.6)	8 (47.1)
Febrile gastroenteritis with dehydration	7 (6.4)	3 (42.9)
Herpes encephalitis	3 (2.8)	2 (66.7)
Bacterial meningitis	1 (0.9)	1 (100)
Metabolic/Toxic causes	18 (16.5)	4 (22.2)
Hyponatremia	13 (11.9)	3 (23.1)
Hypoglycemia	3 (2.8)	0 (0)
Intoxication	3 (2.8)	1 (33.3)
Diabetic ketoacidosis	1 (0.9)	0 (0)
Post-epileptic/Status epilepticus	16 (14.7)	2 (12.5)
Traumatic brain injury	5 (4.6)	1 (20.0)
Other causes	7 (6.4)	2 (28.6)
Unknown etiology	14 (12.8)	1 (7.1)
Malformations	1 (0.9)	0 (0)

### Clinical outcomes

Overall outcomes at hospital discharge were as follows: Survival was 81/109 (74.3%), including 56/81 (69.1% of survivors; 51.4% of total) without sequelae and 25/81 (30.9% of survivors; 22.9% of total) with sequelae. Death occurred in 29/109 (26.6%). Other outcomes were discharged against medical advice in 6/109 (5.5%), transferred to other facility in 3/109 (2.8%), and required mechanical ventilation in 2/109 (1.8%).

The outcome by coma severity (*N* = 109) was as follows. For Stage I (GCS 11–13, *n* = 26), there was 1 death (3.8%) and 25 survivors (96.2%), giving a mortality rate of 3.8%. For Stage II (GCS 9–10, *n* = 49), there were 12 deaths (24.5%) and 37 survivors (75.5%), giving a mortality rate of 24.5%. For Stage III (GCS 6–8, *n* = 22), there were 6 deaths (27.3%) and 16 survivors (72.7%), giving a mortality rate of 27.3%. For Stage IV (GCS 3–5, *n* = 11), there were 11 deaths (100%) and 0 survivors (0%), giving a mortality rate of 100%. For the 1 case not documented, there were 0 deaths (0%) and 1 survivor (100%), giving a mortality rate of 0% (See Table 4). The total was 109 patients with 29 deaths (26.6%) and 81 survivors (74.3%), giving an overall mortality rate of 26.6% (χ²=39.87, *p* < 0.001).

**Table 4:** Outcome according to coma severity (*N* = 109)

Outcome	Stage I (GCS 11–13) *n* = 26	Stage II (GCS 9–10) *n* = 49	Stage III (GCS 6–8) *n* = 22	Stage IV (GCS 3–5) *n* = 11	Total *n* = 109
Survival without sequelae	21 (80.8%)	33 (67.3%)	13 (59.1%)	0 (0%)	67 (61.5%)
Survival with sequelae	5 (19.2%)	16 (32.7%)	9 (40.9%)	0 (0%)	30 (27.5%)
Death	1 (3.8%)	12 (24.5%)	6 (27.3%)	11 (100%)	30 (27.5%)
Transfer	0 (0%)	1 (2.0%)	0 (0%)	0 (0%)	1 (0.9%)
DAMA^a^	0 (0%)	5 (10.2%)	0 (0%)	1 (9.1%)	6 (5.5%)

^a^*DAMA*Discharge against medical advice

**Fig. 3:**
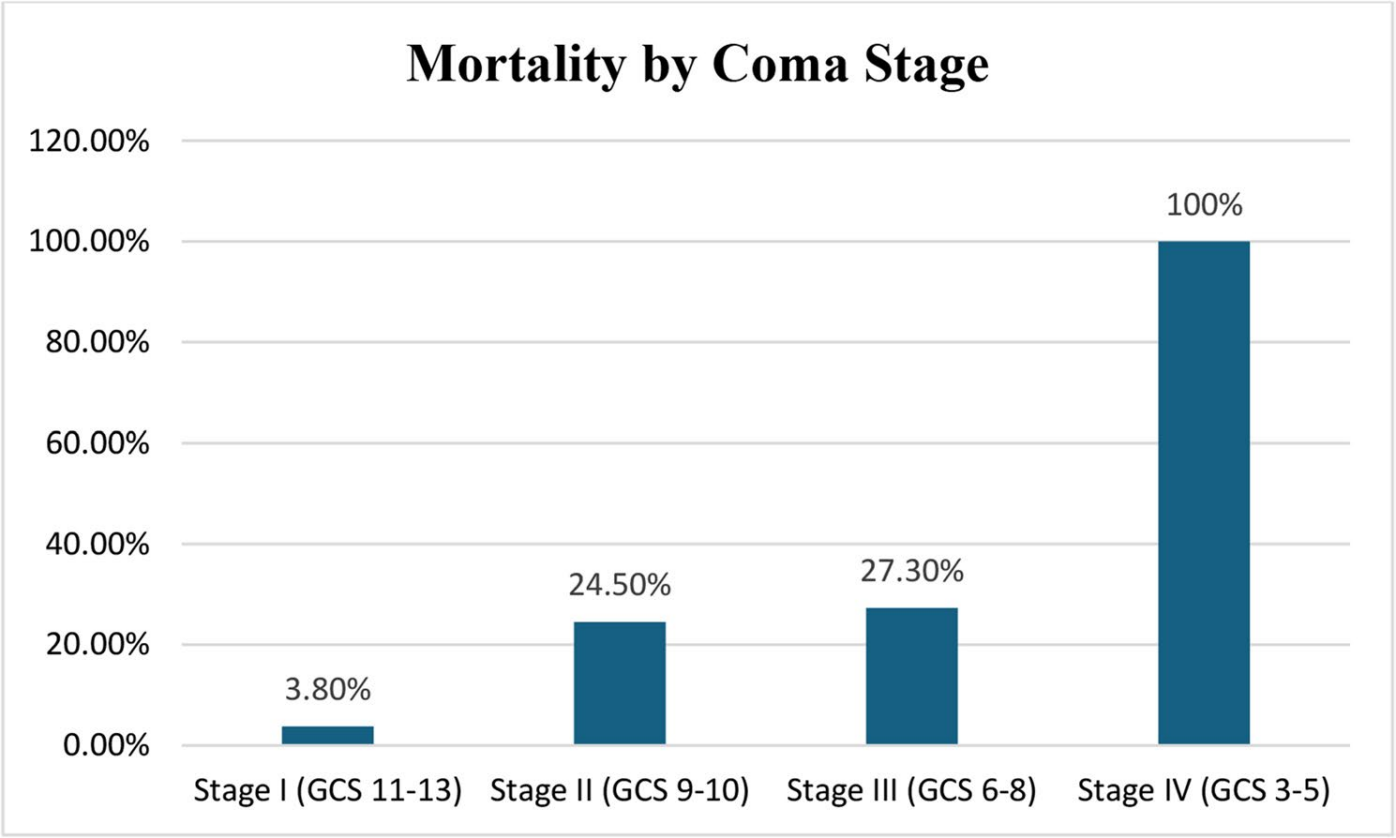
Mortality by coma stage

Figure 3 illustrates mortality rates stratified by coma stage. A striking gradient was observed, with Stage IV (GCS 3–5) demonstrating 100% mortality (11/11 patients), Stage III (GCS 6–8) showing 27.3% mortality (6/22), Stage II (GCS 9–10) demonstrating 24.5% mortality (12/49), and Stage I (GCS 11–13) having the lowest mortality at 3.8% (1/26).

The types of neurological sequelae among survivors (*n* = 81) were as follows: motor deficit in 12 (14.8% of survivors; 11.0% of total cohort), visual impairment in 2 (2.5% of survivors; 1.8% of total), hearing impairment in 2 (2.5% of survivors; 1.8% of total), psychomotor regression in 2 (2.5% of survivors; 1.8% of total), behavioral disorders in 1 (1.2% of survivors; 0.9% of total), and multiple sequelae in 6 (7.4% of survivors; 5.5% of total). The total with any sequela was 25 (30.9% of survivors; 22.9% of total). No sequelae were present in 56 (69.1% of survivors; 51.4% of total) (See Table 5).

**Table 5:** Sequelae among survivors (*n* = 81)

Type of Sequelae		Number, *n* (%)
No sequelae	56 (69.1)	
Any sequelae	25 (30.9)	
Motor deficit	12 (14.8)	
Visual impairment	2 (2.5)	
Hearing impairment	2 (2.5)	
Psychomotor regression	2 (2.5)	
Behavioral disorders	1 (1.2)	

Hospital stay duration was less than 1 week for 57/109 (52.3%), 1 week to 1 month for 13/109 (11.9%), and more than 1 month for 8/109 (7.3%). Twenty-two patients (20.2%) died before completing 1 week, and duration was not documented for 9 patients (8.3%).

### Sensitivity analysis: outcomes by different GCS thresholds

To allow comparison with studies using different coma definitions, we analyzed outcomes using various GCS thresholds. For the threshold of ≤ 14 (our definition), there were 109 patients (12.6% of 864 admissions) with 29 deaths (26.6%), a mortality rate of 26.6%, and sequelae among survivors in 25/81 (30.9%). For the threshold of ≤ 13, there were 83 patients (9.6% of admissions) with 26 deaths (31.3%), a mortality rate of 31.3%, and sequelae among survivors in 19/56 (33.9%). For the threshold of ≤ 10, there were 60 patients (6.9% of admissions) with 22 deaths (36.7%), a mortality rate of 36.7%, and sequelae among survivors in 14/38 (36.8%). For the threshold of ≤ 8 (severe coma), there were 33 patients (3.8% of admissions) with 17 deaths (51.5%), a mortality rate of 51.5%, and sequelae among survivors in 7/16 (43.8%).

This analysis demonstrates that mortality and morbidity rates increase progressively with deeper levels of consciousness impairment, validating the prognostic utility of GCS staging. Using the conventional severe coma definition (GCS ≤ 8), our prevalence of 3.8% is comparable to the 4.8% reported by Ali et al. in Saudi Arabia [9].

### Factors associated with mortality

In the univariate and multivariable analysis of mortality predictors, the following results were found. For age group, among children < 2 years (51 total), there were 20 deaths, with this group serving as the reference. For children 2–5 years (33 total), there were 4 deaths, with an unadjusted OR of 0.17 (95% CI: 0.05–0.59, *p* = 0.005) and an adjusted OR of 0.22 (95% CI: 0.06–0.81, *p* = 0.023). For children > 5 years (25 total), there were 5 deaths, with an unadjusted OR of 0.09 (95% CI: 0.02–0.38, *p* = 0.001) and an adjusted OR of 0.13 (95% CI: 0.03–0.58, *p* = 0.008). For sex, among males (63 total), there were 12 deaths, with this group serving as the reference. Among females (46 total), there were 17 deaths, with an unadjusted OR of 2.48 (95% CI: 1.08–5.68, *p* = 0.032) and an adjusted OR of 2.89 (95% CI: 1.23–6.79, *p* = 0.015). For referral source, among patients referred from a facility (81 total), there were 18 deaths, with this group serving as the reference. Among patients brought directly from home (28 total), there were 11 deaths, with an unadjusted OR of 2.30 (95% CI: 0.96–5.52, *p* = 0.062) and an adjusted OR of 2.76 (95% CI: 1.02–7.47, *p* = 0.046). For city of residence, among patients from Douala (100 total), there were 25 deaths, with this group serving as the reference. Among patients from other cities (9 total), there were 4 deaths, with an unadjusted OR of 0.61 (95% CI: 0.16–2.28, *p* = 0.463); this variable was not included in the final model. For GCS Stage, among patients in Stage I-II (GCS 9–14, 75 total), there were 13 deaths, with this group serving as the reference. Among patients in Stage III-IV (GCS 3–8, 33 total), there were 17 deaths, with an unadjusted OR of 8.67 (95% CI: 3.45–21.78, *p* < 0.001) and an adjusted OR of 6.92 (95% CI: 2.54–18.86, *p* < 0.001). For previous seizures, among patients with no history (90 total), there were 24 deaths, with this group serving as the reference. Among patients with a history (19 total), there were 5 deaths, with an unadjusted OR of 0.95 (95% CI: 0.32–2.86, *p* = 0.931); this variable was not included in the final model. For fever at admission, among patients without fever (29 total), there were 8 deaths, with this group serving as the reference. Among patients with fever (80 total), there were 21 deaths, with an unadjusted OR of 0.89 (95% CI: 0.36–2.23, *p* = 0.807); this variable was not included in the final model (See Table 6)

**Table 6:** Predictors of mortality logistic regression analysis

Variable	Odds Ratio	aOR	95% CI	*p*-value
Age group				
2–5 years vs. <2 years	0.17	0.22	0.05–0.59	**0.017**
> 5 years vs. <2 years	0.09	0.13	0.02–0.38	**0.001**
Sex				
Female vs. Male	3.54	2.89	2.48–4.60	**0.019**
Provenance				
Home vs. Referred	3.24	2.76	2.07–4.42	**0.050**
City of residence				
Outside Douala vs. Douala	0.61	-	0.16–2.28	0.598

Multivariable model characteristics were: Sample size of 109 patients (complete data for all model variables); Hosmer-Lemeshow test: χ²=6.82, df = 8, *p* = 0.556 (good fit); Area under ROC curve: 0.847 (95% CI: 0.774–0.920); Nagelkerke R²: 0.512; and all VIF < 2.5 (no problematic multicollinearity). Key findings from multivariable analysis were that children < 2 years had 4.5–7.7 times higher mortality odds compared to older children; female patients had 2.89 times higher odds of death compared to males (adjusted OR 2.89, 95% CI: 1.23–6.79); direct admission from home was associated with 2.76 times higher mortality odds compared to facility referral; and patients with deep coma (Stage III-IV) had 6.92 times higher mortality odds compared to lighter coma stages.

### Etiology-specific outcomes

Mortality by etiology was as follows. For cerebral malaria (46 total), there were 9 deaths, a mortality rate of 19.6%, and sequelae among survivors in 12/37 (32.4%). For septicemia (17 total), there were 8 deaths, a mortality rate of 47.1%, and sequelae among survivors in 3/9 (33.3%). For gastroenteritis/dehydration (7 total), there was 1 death, a mortality rate of 14.3%, and sequelae among survivors in 1/6 (16.7%). For herpes encephalitis (3 total), there were 2 deaths, a mortality rate of 66.7%, and sequelae among survivors in 1/1 (100%). For bacterial meningitis (1 total), there were 0 deaths, a mortality rate of 0%, and sequelae among survivors in 0/1 (0%). For metabolic/toxic causes (18 total), there were 4 deaths, a mortality rate of 22.2%, and sequelae among survivors in 4/14 (28.6%). For post-epileptic/status epilepticus (16 total), there were 2 deaths, a mortality rate of 12.5%, and sequelae among survivors in 2/14 (14.3%). For traumatic brain injury (5 total), there was 1 death, a mortality rate of 20.0%, and sequelae among survivors in 1/4 (25.0%). For unknown etiology (14 total), there were 3 deaths, a mortality rate of 21.4%, and sequelae among survivors in 2/11 (18.2%). Septicemia and herpes encephalitis demonstrated the highest mortality rates (47.1% and 66.7% respectively).

### Sequelae associated factors

Among survivors (*n* = 81), factors associated with neurological sequelae included hospital stay duration and GCS stage. Regarding hospital stay duration, among those with a stay < 1 week (49 survivors), 12 had sequelae (24.5%). Among those with a stay ≥ 1 week (21 survivors), 11 had sequelae (52.4%). This resulted in an OR of 3.38 (95% CI: 1.21–9.43, *p* = 0.020). Regarding GCS stage, among Stage I-II survivors (62 survivors), 15 had sequelae (24.2%). Among Stage III survivors (16 survivors), 10 had sequelae (62.5%). This resulted in an OR of 5.22 (95% CI: 1.62–16.83, *p* = 0.005). Note that all Stage IV patients died; no survivors were available for comparison.

## Discussion

This cross-sectional study with retrospective and prospective components provides comprehensive data on the epidemiology, etiology, and outcomes of pediatric coma at a major tertiary referral center in Douala, Cameroon. The 12.6% prevalence of coma among hospitalized children is consistent with previous reports suggesting that coma accounts for 10–15% of pediatric hospitalizations [5–7], though it exceeds the 5.4% reported in Lomé, Togo [3] and falls below the 14.4% found in Cairo, Egypt [1]. These variations likely reflect differences in referral patterns, healthcare-seeking behavior, disease burden, and coma definitions across different African settings.

### Demographic characteristics

The male predominance (sex ratio 1.4:1) observed in our cohort is consistent with several previous studies [1, 3, 8]. The mean age of 48.8 months and the predominance of children under 5 years (64.2%) align with global patterns showing that younger children are at higher risk for coma. This age-related vulnerability likely reflects multiple factors including: (1) immature cerebral physiology and autoregulation, (2) higher susceptibility to infections, (3) increased risk of metabolic derangements, and (4) limited physiological reserves [5, 14].

The high proportion of referrals from other healthcare facilities (74.3%) underscores DGOPH’s role as a tertiary referral center for the Douala metropolitan area and surrounding regions. Interestingly, direct admission from home was independently associated with significantly higher mortality (adjusted OR 2.76, 95% CI: 1.02–7.47). This association possibly reflects: (1) delays in care-seeking with more advanced disease at presentation, (2) lack of stabilization and initial management before arrival, (3) more fulminant disease courses, or (4) socioeconomic factors affecting healthcare access. This finding emphasizes the critical importance of efficient referral systems, community health education about recognizing danger signs in children, and strengthening primary care capabilities for initial stabilization.

### Clinical presentation and severity

The distribution of coma severity showed that most patients presented with moderate coma severity: 45.0% in Stage II (GCS 9–10) and 23.9% in Stage I (GCS 11–13). Severe deep coma (Stage IV, GCS 3–5) occurred in 10.1%. Our sensitivity analysis demonstrated that using the conventional severe coma definition (GCS ≤ 8) yields a prevalence of 3.8%, comparable to the 4.8% reported by Ali et al. in Saudi Arabia [9], validating our broader definition while allowing international comparisons.

The strong relationship between coma severity and outcome is evident from the striking mortality gradient: Stage IV demonstrated 100% mortality (11/11 patients), Stage III 27.3% (6/22), Stage II 24.5% (12/49), and Stage I only 3.8% (1/26). These findings are consistent with numerous studies demonstrating that GCS score is a powerful predictor of outcome in pediatric coma [11–13]. The 100% mortality in Stage IV coma (GCS 3–5) reflects both the severity of underlying brain dysfunction and limitations in advanced life support capabilities at our center during the study period.

Seizures at admission were present in 68.8% of patients, predominantly generalized (85.3%). This high frequency likely reflects both the etiologies prevalent in our setting (particularly cerebral malaria, which commonly presents with seizures, and status epilepticus) and the pathophysiological mechanisms of coma in children, where seizure activity frequently accompanies acute brain dysfunction.

### Etiological profile

The predominance of infectious causes (62.4%) in our study mirrors findings from other sub-Saharan African and Asian countries, where infections remain the leading cause of pediatric coma [1, 3, 8–10]. However, the specific distribution of infectious etiologies reflects regional epidemiology and healthcare context.

#### Cerebral malaria

Emerged as the single most common cause (42.2%, 46/109), consistent with the high malaria endemicity in Cameroon and the location of Douala in the coastal tropical region. This finding aligns with studies from other malaria-endemic regions where cerebral malaria accounts for 30–50% of pediatric coma cases. The 19.6% mortality rate (9/46 cases) for cerebral malaria in our study falls within the reported range of 15–25% for this condition [3, 8], though it remains unacceptably high given that malaria is both preventable and treatable. The neurological sequelae rate of 32.4% among cerebral malaria survivors emphasizes the substantial morbidity burden beyond mortality.

#### Septicemia 

(15.6%, 17/109) was the second most common infectious cause, with a notably high mortality rate of 47.1% (8/17). This high fatality rate likely reflects several factors: (1) severity of sepsis at presentation with delayed care-seeking, (2) limited intensive care capabilities (no dedicated pediatric ICU, limited mechanical ventilation), (3) potential antimicrobial resistance, and (4) challenges in identifying specific pathogens to guide therapy (blood cultures performed in only 16.5% due to resource constraints).

The low frequency of *bacterial meningitis* (0.9%, 1/109) requires careful interpretation. This may be attributable to several factors: (1) widespread vaccination against *Haemophilus influenzae* type b and *Streptococcus pneumoniae* through Cameroon’s Expanded Programme on Immunization, (2) empirical antibiotic use before lumbar puncture (common practice in our setting when meningitis is suspected), (3) diagnostic limitations (only 31.2% of patients underwent lumbar puncture due to contraindications, parental refusal, or clinical judgment that LP was unnecessary), or (4) genuine low incidence in our specific population during the study period.

#### Febrile gastroenteritis with dehydration 

(6.4%, 7/109) represents a preventable cause of coma that could be addressed through improved community management of diarrheal diseases, oral rehydration therapy promotion, and timely healthcare facility presentation.

#### Metabolic and toxic causes 

(16.5%, 18/109) represented the second most common etiological category. *Hyponatremia* (11.9%, 13/109) was the most frequent metabolic disturbance, consistent with reports that children are at higher risk than adults for hyponatremic encephalopathy due to their relatively larger brain-to-intracranial volume ratio and reduced adaptive capacity [15–17]. Many hyponatremia cases were secondary to gastroenteritis with inappropriate fluid management. *Hypoglycemia *(2.8%, 3/109) was less common than expected, possibly due to systematic capillary glucose monitoring and immediate correction in the emergency department before patient enrollment. *Intoxications *(2.8%, 3/109) included petroleum product ingestions and medication overdoses, highlighting the need for prevention education and poison control services.

#### Post-epileptic coma and status epilepticus 

(14.7%, 16/109) constituted a significant proportion, reflecting the high burden of seizure disorders in African children [18–21]. The fact that only 5.5% (6/109) of our cohort had known epilepsy suggests that many cases represented first presentations of seizure disorders or seizures secondary to acute illnesses (particularly febrile seizures or infection-related seizures). The relatively low mortality (12.5%) in this category compared to infectious causes emphasizes the importance of prompt anticonvulsant therapy and airway management.

#### Traumatic brain injury

(4.6%, 5/109) was relatively uncommon in our series. This low proportion contrasts with data from high-income countries where trauma often accounts for 20–30% of pediatric coma cases [22–24]. The low frequency in our study likely reflects several factors: (1) severe trauma cases may be managed at other facilities with surgical capabilities, (2) rapid pre-hospital mortality for the most severe cases, (3) parental delays in seeking care for trauma, or (4) genuine lower incidence of severe pediatric trauma in our catchment area.

The *12.8% of cases with unknown etiology* despite clinical and laboratory investigation highlights diagnostic challenges in resource-limited settings, including: (1) limited access to advanced neuroimaging (CT in only 22.0%, MRI in 4.6%), (2) restricted microbiological diagnostics (blood cultures in 16.5%, CSF analysis in 31.2%), (3) unavailability of specialized metabolic testing (ammonia, lactate, amino acids, organic acids), (4) limited toxicology screening, and (5) no access to viral PCR or antibody testing beyond herpes. Some of these “unknown” cases may have represented viral encephalitides, autoimmune encephalitis, or rare metabolic disorders that could not be diagnosed with available resources.

### Diagnostic approach and resource limitations

The diagnostic workup in our study reflected typical resource constraints of many African healthcare settings. While basic investigations (capillary glucose 98.2%, malaria testing 89.0%, hemoglobin 96.3%) were performed in most patients, more advanced studies were limited: brain imaging in only 26.6%, lumbar puncture in 31.2%, and EEG in 8.3%.

The *low rate of lumbar puncture *(31.2%) is concerning given the need to exclude treatable causes such as bacterial meningitis. Barriers included: (1) concerns about safety in patients with altered consciousness and potential raised intracranial pressure, (2) lack of CT availability to exclude contraindications before LP, (3) empirical antibiotic treatment initiated before the procedure, (4) parental refusal in some cases, and (5) clinical judgment that LP was unnecessary when alternative diagnosis was clear (e.g., definite cerebral malaria). This limitation may have led to underdiagnosis of CNS infections.

#### Neuroimaging 

Was severely limited by cost (not covered by the national health insurance) and availability (single CT scanner shared across all hospital services, no MRI in our facility). The 22.0% CT rate primarily included patients with suspected traumatic brain injury, focal neurological signs, or when families could afford the out-of-pocket cost. This imaging limitation likely affected: (1) missed diagnoses of structural lesions, (2) inability to definitively exclude raised intracranial pressure before LP, and (3) delayed recognition of complications like cerebral edema or hemorrhage.

These diagnostic limitations emphasize the need for: (1) strengthened basic laboratory services, (2) improved access to neuroimaging through insurance coverage and equipment availability, (3) development of clinical decision rules for resource-appropriate investigation, and (4) point-of-care diagnostics for common pathogens.

### Outcomes and prognostic factors

The overall mortality rate of 26.6% in our study falls within the wide range (3–84%) reported globally for pediatric coma [8], but substantially exceeds rates from high-income countries (typically 5–15%) and is comparable to other resource-limited settings in Africa (27.6% in Lomé [3], 50% in Cairo [1]). This mortality reflects both the severity of presenting illnesses and systemic healthcare limitations including: (1) delays in presentation and referral, (2) limited intensive care capabilities, (3) diagnostic limitations affecting targeted therapy, (4) resource constraints for optimal supportive care, and (5) high burden of severe preventable diseases.

### Independent predictors of mortality

Our multivariable analysis (controlling for confounding) identified several independent predictors of mortality:

#### Age < 2 years

Infants and young toddlers had 4.5–7.7 times higher mortality odds compared to older children (adjusted OR for age 2–5 years: 0.22; for age > 5 years: 0.13). This age-related vulnerability reflects multiple factors: immature cerebral autoregulation and blood-brain barrier, limited physiological reserves (smaller glycogen stores, reduced ability to maintain homeostasis), higher susceptibility to hypoglycemia and electrolyte disturbances, greater vulnerability to infection-related complications and difficulty assessing clinical status and response to treatment in pre-verbal children.

#### Female sex

The unexpected finding of higher mortality in females (adjusted OR 2.89, 95% CI: 1.23–6.79) contrasts with some previous studies showing male predominance in poor outcomes. This finding requires further investigation but could reflect: (1) sex-specific differences in care-seeking behavior (possible delayed presentation for female children), (2) underlying nutritional status differences, (3) biological factors (hormonal, genetic), or (4) chance finding in our limited sample size. This observation warrants validation in larger multi-center studies and investigation of potential mechanisms.

#### Direct admission from home

Children brought directly from home had 2.76 times higher mortality odds (95% CI: 1.02–7.47) compared to those referred from other healthcare facilities. This association suggests several possible mechanisms: (1) delay in initial care-seeking leading to more advanced disease, (2) lack of stabilization and initial management (IV access, glucose correction, initial antibiotics, seizure control) before arrival at tertiary center, (3) more fulminant disease courses prompting direct presentation, or (4) socioeconomic factors affecting healthcare access and quality. This finding emphasizes the critical importance of: (a) community health education about recognizing pediatric danger signs, (b) strengthening primary care facilities’ capabilities for emergency stabilization, (c) efficient referral systems with appropriate transfer protocols, and (d) addressing socioeconomic barriers to healthcare access.

#### Deeper coma stages

Patients with deep coma (Stage III-IV, GCS 3–8) had 6.92 times higher mortality odds (95% CI: 2.54–18.86) compared to lighter coma stages (Stage I-II, GCS 9–14). The 100% mortality in Stage IV coma (GCS 3–5) reflects both the severity of underlying brain dysfunction and the limited reversibility of such profound depression of consciousness with available intensive care resources. This strong relationship between GCS and outcome has been consistently demonstrated across multiple studies and settings [11–13], validating the prognostic utility of systematic coma severity assessment.

### Neurological sequelae among survivors

Among survivors, 30.9% (25/81) had neurological sequelae at hospital discharge, predominantly motor deficits (14.8%, 12/81 survivors). This substantial morbidity burden emphasizes that mortality alone underestimates the total disease burden of pediatric coma. The true burden is likely even higher as: (1) our assessment was at hospital discharge only (median 6 days), missing delayed sequelae that may emerge weeks to months later, (2) clinical assessment alone may miss subtle deficits, and (3) longer follow-up often reveals additional functional impairments affecting quality of life and development.

Factors associated with higher sequelae risk included: longer hospitalization (> 1 week: OR 3.38), deeper coma stages (Stage III survivors: 62.5% vs. Stage I-II: 24.2%, OR 5.22) and specific etiologies (herpes encephalitis 100%, cerebral malaria 32.4%).

These findings underscore the need for: (1) long-term follow-up programs for all survivors (at 3, 6, 12 months and beyond), (2) early identification and intervention for developmental delays, (3) rehabilitation services (physical therapy, occupational therapy, speech therapy), (4) educational support for children with cognitive sequelae, and (5) family counseling and support services.

#### Resource limitations and ICU context

The very low ICU admission rate (1.8%, 2/109) relative to mortality (26.6%) requires important contextual explanation. During the study period, DGOPH had severely limited intensive care capabilities: there was no dedicated pediatric ICU unit, with critically ill children managed in a high-dependency area within the general pediatric ward. Available resources were limited to two pediatric ventilators for the entire hospital, basic non-invasive monitoring, and limited availability of inotropic support, with no capacity for advanced interventions. The implications were that most critically ill children requiring intensive care were managed in the general ward with high nurse-to-patient ratios, not in an ICU setting. The high mortality despite low formal ICU admission reflects both the severity of illness at presentation and profound resource limitations in providing potentially life-saving advanced supportive care. This context is crucial for interpreting outcomes and highlights the urgent need for enhanced critical care infrastructure, including dedicated pediatric ICU beds, trained staff, and essential equipment in similar resource-limited settings.

#### Clinical and public health implications

Our findings have important implications for clinical practice and public health policy in Cameroon and similar resource-limited settings. Cerebral malaria constitutes the largest single preventable cause of pediatric coma (42.2%), necessitating strengthened malaria prevention and control strategies. The association between direct home admission and increased mortality emphasizes the need for community awareness programs and early recognition of pediatric danger signs. Improved outcomes for referred patients suggest that referral systems, while functional, require enhancement with standardized protocols and emergency transport. Reducing the high rate of unknown etiology requires improved access to neuroimaging and enhanced diagnostic capabilities. The high mortality in severe cases underscores the urgent need for dedicated pediatric ICU development with appropriate staffing and equipment. The significant rate of neurological sequelae among survivors necessitates the establishment of long-term follow-up and accessible rehabilitation services. Future research priorities should focus on multi-center prospective cohorts, long-term outcome studies, and implementation research on prevention strategies adapted to resource-limited settings.

#### Study strengths

This study has several important strengths, including an adequate sample size exceeding calculated requirements and comprehensive data collection. The dual methodology combining retrospective and prospective approaches maximized case identification, while systematic etiological classification used standardized diagnostic criteria. The multivariable analysis identified independent predictors while controlling for confounding, and sensitivity analyses allowed comparison with studies using different coma definitions. The study provides clinically relevant, actionable data for policy and practice improvement, with transparent reporting of limitations and resource constraints.

#### Study limitations

This study has several important limitations to consider. Design limitations include its single-center nature, which limits generalizability, and the cross-sectional design with outcome assessment only at hospital discharge, without post-discharge follow-up to capture late mortality or delayed sequelae. Diagnostic limitations were significant, with limited access to advanced diagnostics like neuroimaging, lumbar puncture, and metabolic screening, which likely contributed to the 12.8% unknown etiology rate. The assessment of neurological sequelae was clinical without formal testing tools, potentially missing subtle deficits. Sample considerations include possible selection bias as a tertiary referral center, and variable-specific limitations such as incomplete data for some measures. The effects of resource constraints introduced variability in management and likely influenced outcomes. Statistical limitations include limited power for some subgroup analyses. External validity is a concern due to the single urban center setting and Cameroon-specific epidemiology. Finally, several potential confounders, such as socioeconomic status and pre-hospital care quality, were not systematically measured. These limitations emphasize the need for future multi-center prospective studies with longer follow-up, standardized protocols, and comprehensive assessment tools.

## Conclusion

This cross-sectional study demonstrates that pediatric coma at a tertiary hospital in Douala, Cameroon predominantly affects young children (mean age 48.8 months, 64.2% under 5 years) and is primarily attributable to infectious causes (62.4%), particularly cerebral malaria (42.2%). The mortality rate of 26.6% remains unacceptably high, with several independent predictors identified through multivariable analysis: younger age (< 2 years: 4.5–7.7 times higher odds), female sex (2.89 times higher odds), direct admission from home (2.76 times higher odds), and deeper coma stages (Stage III-IV: 6.92 times higher odds).

Among survivors, nearly one-third (30.9%) experience neurological sequelae at discharge, predominantly motor deficits (14.8%), with additional burdens of visual impairment, hearing impairment, psychomotor regression, and behavioral disorders. The total disease burden thus extends far beyond mortality alone.

This study underscores several critical, actionable priorities for mitigating the high mortality and morbidity from pediatric coma in Central Africa. Essential public health measures include the urgent scale-up of malaria control programs, alongside widespread community education to improve early recognition of danger signs and reduce delays in seeking care. Within the health system, immediate efforts must focus on strengthening referral pathways, enhancing diagnostic capacity with essential imaging and point-of-care tests, and developing dedicated pediatric intensive care with appropriate staffing and equipment.

To address the long-term consequences for survivors, establishing structured follow-up and rehabilitation programs is imperative. Concurrently, strategic research investment in multi-center, prospective studies with extended follow-up is needed to generate robust evidence, fully characterize long-term outcomes, and effectively evaluate these proposed interventions.

Early recognition and prompt, appropriate management of preventable causes—particularly cerebral malaria, sepsis, and metabolic derangements—could substantially reduce both mortality and morbidity from pediatric coma. Addressing the identified risk factors (young age vulnerability, delayed presentation from home, lack of intensive care capabilities) through targeted interventions represents a critical pathway toward improved outcomes.

Future multi-center prospective studies with longer follow-up periods (minimum 12 months), standardized diagnostic protocols, comprehensive neurodevelopmental assessments, and systematic collection of socioeconomic variables are needed to fully characterize the spectrum and long-term outcomes of pediatric coma in this region and to evaluate the effectiveness of proposed interventions.

## Supplementary Information

Supplementary Material 1

## Data Availability

The datasets generated and analyzed during the current study are not publicly available due to ethical restrictions and privacy protection requirements for pediatric medical data. However, de-identified datasets are available from the corresponding author (enyamad@yahoo.fr) on reasonable request and with appropriate ethical approvals from requesting institutions.

## Abbreviations

ARAF: Ascending Reticular Activating Formation
CI: Confidence Interval
CRP: C-Reactive Protein
CSF: Cerebrospinal Fluid
CSPro: Census and Survey Processing System
CT: Computed Tomography
DAMA: Discharge Against Medical Advice
EEG: Electroencephalography
ENT: Ear, Nose, and Throat
GCS: Glasgow Coma Scale
Hb: Hemoglobin
DGOPH: Douala Gyneco-Obstetric and Pediatric Hospital
ICU: Intensive Care Unit
MNAR: Missing Not At Random
MRI: Magnetic Resonance Imaging
OR: Odds Ratio
RDT: Rapid Diagnostic Test
ROC: Receiver Operating Characteristic
SD: Standard Deviation
SPSS: Statistical Package for the Social Sciences
VIF: Variance Inflation Factor
WBC: White Blood Cell
WHO: World Health Organization
